# Two myxobacterial strains against *Rhizoctonia solani*: comparative genomics of antagonistic potential and phenotypic evaluation of growth-promoting and disease-control effects

**DOI:** 10.3389/fmicb.2026.1975171

**Published:** 2026-10-14

**Authors:** Youkai Gao, Yihao Fu, Chengyu Jin, Jianan Cheng, Yuguang Wang, Yan Sun, Gui Geng, Yanchun Sun

**Affiliations:** College of Advanced Agriculture and Ecological Environment, Heilongjiang University, Harbin, China

**Keywords:** continuous cropping obstacle, *Cystobacter ferrugineus*, *Cystobacter fuscus*, myxobacteria, sugar beet, whole-genome sequencing

## Abstract

**Introduction:**

Although sugar beet (*Beta vulgaris s*sp. *vulgaris*) is a major sugar crop in China, continuous cropping obstacles frequently constrain its productivity and quality. The application of antagonistic microorganisms or plant growth-promoting rhizobacteria (PGPR) offers a promising biological method for alleviating these constraints. This study evaluated two previously isolated myxobacterial strains with documented antagonistic activity against *Rhizoctonia solani*: *Cystobacter ferrugineus* W14 and *Cystobacter fuscus* W11.

**Methods:**

Their effects on sugar beet seedling growth under continuous cropping were assessed in a pot experiment, and whole-genome sequencing enabled the comparative profiling of disease resistance-related genes and functional pathways.

**Results:**

Inoculation with either strain significantly increased seedling growth parameters, including stem diameter, plant height, and leaf fresh weight, compared with the control (CK). Specifically, strains W14 and W11 significantly increased plant height by 38.40 and 33.62%, respectively, and stem diameter by 44.54 and 31.93%, respectively. Moreover, disease incidence was markedly reduced. W14 and W11 yielded disease index values of 16.11 and 19.46, respectively, corresponding to significant reductions of 70.49 and 64.35%, respectively, compared with the CK. Whole-genome sequencing revealed that strain W14 contained more GH19 family chitinase genes, GH5 family glucanase genes, and secondary metabolite biosynthetic gene clusters than strain W11. Functional annotation assigned more strain W14 genes to three major antagonism-related categories: cell wall degradation, antibiotic synthesis, and signal transduction. Gene Ontology (GO) and Kyoto Encyclopedia of Genes and Genomes (KEGG) pathway analyses consistently identified more genes involved in metabolic processes and secondary metabolite biosynthesis pathways in strain W14 than in strain W11.

**Conclusion:**

Both myxobacterial strains promoted sugar beet seedling growth and protected against diseases associated with continuous cropping, whereas strain W14 showed a greater genomic capacity for antagonistic traits. These findings provide a theoretical basis for the development of novel biocontrol agents. These genomic differences represent candidate correlates of antagonistic potential and require further functional validation.

## 1 Introduction

Sugar beet (*Beta vulgaris s*sp. *vulgaris*) is a major temperate crop that can supply nearly 30% of global sugar production from its tuberous roots, while its foliage is used to produce bioethanol, biofuels, and animal feed (Dohm et al., 2013; Li et al., 2022). Its deep taproot system and high nutrient requirements make sugar beet particularly vulnerable to continuous cropping. By disrupting the equilibrium of soil microbial communities and aggravating soilborne diseases, prolonged monoculture reduces both sucrose content and yield, effects collectively termed continuous cropping obstacles (Cui et al., 2022; Zhang et al., 2022). A prevalent soilborne fungal disease in continuously cropped sugar beet fields is damping-off and root rot caused by *Rhizoctonia solani*, which seriously threatens sustainable production (Abbas et al., 2022; Farhaoui et al., 2024; Le Cointe et al., 2016). Accordingly, screening microorganisms for biocontrol or plant growth-promoting activity and developing them as antagonistic agents or PGPR has become an important green management strategy (Morales-Cedeño et al., 2021).

Myxobacteria (phylum Proteobacteria and class Deltaproteobacteria) are Gram-negative bacteria with complex social behaviors. Rod-shaped vegetative cells move by gliding motility, allowing them to actively prey on other microorganisms (Dawid, 2000; Reichenbach, 1999; Wang et al., 2020). This predatory lifestyle relies on the secretion of diverse lytic enzymes, including chitinases and glucanases, and secondary metabolites that efficiently degrade fungal hyphae and suppress pathogen growth (Cao et al., 2015; Phillips et al., 2022). These properties make myxobacteria a promising candidate for the biological control of plant diseases. For instance, strains belonging to *Myxococcus* and *Corallococcus* strongly antagonize soilborne phytopathogenic fungi, including *R. solani*, which causes rice sheath blight, and *Fusarium oxysporum*, and have effectively decreased disease incidence in pot experiments (Dahm et al., 2015; Li et al., 2017). Moreover, some myxobacteria may support a healthy plant–soil microecosystem through nutrient competition and community modulation (Xia et al., 2023).

However, most research has focused on *Myxococcus* and *Corallococcus*, whereas *Cystobacter* has received comparatively little attention. Whether the antagonistic and growth-promoting capacities differ among *Cystobacter* species and which genomic features underlie such variations remain unclear. In a previous study, we isolated two *Cystobacter* strains, *C. fuscus* W11 and *C. ferrugineus* W14, from the rhizosphere soil of continuously cropped sugar beet and demonstrated their antagonistic activity against *R. solani*. Preliminary plate assays revealed varying levels of inhibition, with strain W14 exerting stronger antifungal activity. This phenotypic divergence raises the possibility that the strains differ in genes encoding cell wall-degrading enzymes, such as chitinases and glucanases, and in secondary metabolite biosynthetic gene clusters. Therefore, comparative genomics may reveal the molecular determinants of their antagonistic potential and guide the rational selection of highly effective biocontrol strains.

In the present study, we investigated strains W11 and W14 with three objectives: (1) to assess their growth-promoting effects and efficacy against *R. solani*-associated disease under continuous cropping by conducting a sugar beet pot experiment; (2) to perform whole-genome sequencing of both strains using third-generation technologies and compare them genomically; and (3) to annotate gene functions with the GO and KEGG databases, predict secondary metabolite biosynthetic gene clusters using antiSMASH v5.0.0, and examine cell wall-degrading enzyme genes and pathogen–host interaction-related genes using the CAZy and PHI databases, respectively. By integrating different genomic annotation approaches, this study aimed to explore potential genomic correlates of the differences in antagonistic potential and to identify candidate genes for future functional validation. These findings expand the repertoire of biocontrol agents available for continuously cropped sugar beet and support the development of myxobacterium-based bioformulations.

## 2 Materials and methods

### 2.1 Experimental materials and design

Sugar beet cultivar ‘KWS1260’ (KWS GmbH, Einbeck, Germany) was used as the plant material. *Cystobacter fuscus* W11 and *Cystobacter ferrugineus* W14, previously isolated and maintained in our laboratory, were selected because preliminary tests revealed that both strains produced siderophores, whereas only strain W14 solubilized phosphate. Dual-culture plate assays further showed that both strains were antagonistic to *R. solani*. Potato dextrose agar (PDA), purchased from Beijing Aoboxing Biotechnology Co., Ltd. (China), was used as the culture medium. The tested soils were collected from a black soil region in the Hulan District, Harbin, Heilongjiang Province (46°00′14″E, 126°38′49″N). They comprised soil from a two-year continuous sugar beet cropping system with a history of *R. solani* infestation and soil from a soybean rotation system. Field water and fertilizer management were consistent across the two soil types. Soil samples obtained using the five-point sampling method were thoroughly mixed. The experiment was performed in a controlled environment light culture room at the College of Agriculture, Heilongjiang University.

The pot experiment consisted of four treatments: soybean-cropped soil as a positive rotation control (NC), soil continuously cropped with sugar beet for 2 years as a negative control (CK), continuously cropped soil inoculated with strain W11 suspension (OD_600_ = 1.0) (W11), and the same soil inoculated with strain W14 suspension (OD_600_ = 1.0) (W14). Each treatment had nine replicates. Preliminary experiments identified these OD_600_ values as optimal for antagonistic activity.

Each pot received 900 g of homogenized soil and was sown with ‘KWS1260’ seeds. Following emergence, the seedlings were thinned to five per pot. Plants were maintained under a light intensity of 700 μmol m^−2^ s^−1^ and a 14 h light/10 h dark photoperiod. Day and night temperatures were (28 ± 1)°C and (21 ± 1)°C, respectively, with a relative humidity of 40–50%. On days 7, 14, and 21 after sowing, a sterile syringe was used to apply 5 mL of the corresponding bacterial suspension to each seedling by root irrigation into the rhizosphere, 1–2 cm from the stem base.

Detailed information on the isolation, purification, and identification of strains W11 and W14 is provided in the Supplementary material.

### 2.2 Strain activation and preparation of bacterial suspensions

After storage at −80 °C, strains W11 and W14 were thawed at room temperature. Under a sterile laminar-flow hood, fruiting bodies were collected using sterilized toothpicks, inoculated onto VY/2 solid medium, and incubated at 30 °C for revival. Subsequently, well-grown colonies were transferred to fresh VY/2 plates and cultured at 30 °C for expansion. Single colonies collected from each culture margin were inoculated into VY/2 liquid medium and grown in a shaking incubator (Model ZWYR-2402, Shanghai Zhicheng Analysis Instrument Manufacturing Co., Ltd., Shanghai, China) at 30 °C and 180 rpm for 7 d. After centrifugation at 5000 rpm, the supernatants were discarded, and the cell pellets were resuspended in sterile distilled water. A spectrophotometer was used to adjust the suspensions to the required OD_600_.

### 2.3 Measurement of plant growth parameters

Eight uniform seedlings were randomly selected from each treatment as biological replicates. Plant height and root length were measured using a tape measure. Stem diameter was measured using a Vernier caliper (0.02 mm precision), and leaf and root fresh and dry weights were measured using an electronic analytical balance (Hangzhou Wante Weighing Apparatus Co., Ltd., model 5,001). For dry weight determination, the samples were oven-dried at 105 °C for 30 min to inactivate enzymes and at 75 °C to a constant weight.

### 2.4 Determination of plant disease index and strain inhibition rate

Disease symptoms were monitored throughout seedling growth. No artificial inoculation with *R. solani* was performed. Disease developed naturally in the continuously cropped soil with a history of *R. solani* infestation. Therefore, the disease index reflects natural disease incidence under these experimental conditions. A disease severity scale (Table 1) was established, and the disease index was calculated using Equation 1. The inhibition rate was calculated using Equation 2.


$$\begin{array}{l} \text{Disease Index}\\ =\frac{\sum (\text{Number of diseased plants in each grade}\times \text{Grade value})}{\text{Total number of plants}\times \text{Highest grade value}}\times 100 \end{array}$$ (1)

$$\text{Inhibition rate}(\%)=\frac{(D_{c}-D_{t})\times 100\%}{D_{c}-D_{0}}$$ (2)

**Table 1:** Disease severity rating scale for sugar beet damping-off.

Grade	Description
0	Healthy plant with no visible symptoms
1	Small, isolated brown lesions on the roots or stem base without tissue constriction
2	Enlarged, coalescing lesions forming distinct brown rings with slight constriction of the stem base or taproot
3	Severe constriction of the stem base or extensive dark-brown rot of the taproot
4	Complete stem-base rot with severe constriction, or extensive root rot
5	Plant dead

Note: D_c_ denotes the average diameter of the pathogen colony in the control group, D_t_ denotes the average diameter of the pathogen colony in the confrontation group, and D_0_ denotes the diameter of the inoculum plug (6 mm).

### 2.5 Whole-genome sequencing and assembly

Fruiting bodies collected from fresh cultures were submitted to General Biosystems Co., Ltd. (Anhui, China) for complete genome sequencing on Oxford Nanopore Technologies (ONT) platform. Genomic DNA was extracted using the CTAB method with a QIAGEN Genomic-tip 20/G kit, and its quality was evaluated using NanoDrop, Qubit, and agarose gel electrophoresis. High-quality DNA was fragmented using g-TUBE and subjected to end repair, A-tailing, barcode ligation, and sequencing adapter ligation to construct libraries, which were sequenced on a PromethION 48 sequencer for 72 h. Raw reads were base-called with Guppy v3.2.6 and converted to FASTQ format, followed by the removal of low-quality reads and fragments shorter than 2000 bp. Clean reads were assembled using Canu v1.5, and BRIG software was used to circularize and annotate the assembled contigs. Secondary metabolite biosynthetic gene clusters were predicted using antiSMASH v5.0.0. Predicted protein sequences were aligned against the NCBI Nr and KEGG databases using BLAST (e-value: 1e−5), and GO terms were annotated using Blast2GO. In addition, the CAZy and PHI databases were searched for genes associated with cell wall degradation and pathogen–host interactions. These database searches and predictions collectively served as different genomic annotation approaches to identify candidate genes and pathways related to antagonism, rather than independent multi-omics validations.

### 2.6 Statistical analysis

Plant growth parameters were subjected to one-way analysis of variance (ANOVA) using IBM SPSS 27 (SPSS Inc., Somers, USA), followed by Tukey’s HSD test for multiple comparisons (*p* < 0.05) at a 95% confidence level. Graphs were generated using GraphPad Prism, and schematic illustrations were created using BioRender.

## 3 Results

### 3.1 Growth parameters

Figure 1 and Table 2 show the effects of the different *Cystobacter* suspension treatments on sugar beet seedling growth. As shown in Figure 1A, seedlings were less vigorous under continuous cropping (CK) than under rotation (NC). However, inoculation with W11 or W14 markedly enhanced the growth of continuously cropped sugar beet seedlings, and certain parameters exceeded those of the rotation treatment. Compared with NC, CK reduced plant height (Height), stem diameter (SD), aboveground fresh and dry weights (FW and DW), root length (RL), and root fresh and dry weights (RFW and RDW). Plant height and stem diameter declined significantly by 10.53 and 11.52%, respectively. Bacterial suspensions effectively alleviated the adverse effects of continuous cropping obstacles. Compared with CK, W11 significantly increased plant height, stem diameter, and root length by approximately 33.62%, 31.93%, and 10.22%, respectively, whereas the corresponding increases under W14 were 38.40%, 44.54%, and 17.84%. Furthermore, W11 and W14 produced significantly greater aboveground and root fresh weights than NC. Although W14 generally showed higher mean values than W11 for most growth parameters, significant differences between W11 and W14 were detected only for stem diameter and root fresh weight, being 9.55 and 34.29% greater, respectively, under W14 than under W11 (Tukey’s HSD, *p* < 0.05). For the remaining growth parameters, including plant height, aboveground fresh weight, aboveground dry weight, root length, and root dry weight, no significant differences were detected between W11 and W14 (Tukey’s HSD, *p* > 0.05). However, both W11 and W14 differed significantly from CK for most parameters, as indicated by the letter annotations in Table 2. Complete pairwise comparisons are provided in Table 2.

**Figure 1:**
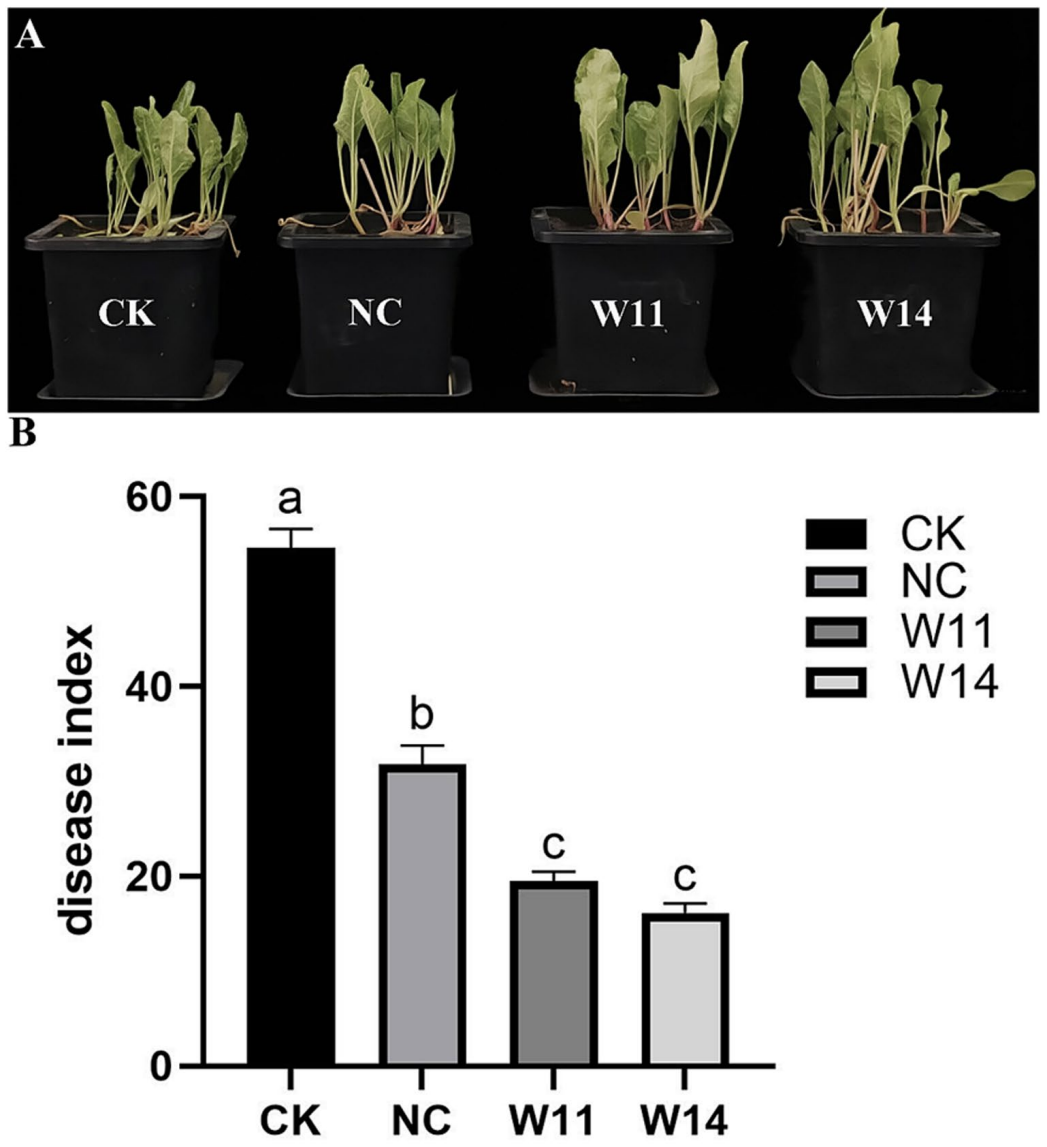
Effects of the two myxobacterial strains on the growth and disease index of sugar beet seedlings. **(A)** Representative phenotypes of sugar beet seedlings under different treatments. **(B)** Effects of different treatments on the disease index of sugar beet seedlings. Data in **(B)** are presented as mean ± standard deviation. Different lowercase letters above the bars in **(B)** indicate significant differences among treatments (Tukey’s HSD test, *p* < 0.05). NC, soil from a soybean rotation system; CK, soil under continuous sugar beet cropping for 2 years; W11, continuously cropped soil (2 years) inoculated with strain W11; W14, continuously cropped soil (2 years) inoculated with strain W14. These abbreviations are used throughout the following figures.

**Table 2:** Growth parameters of sugar beet seedlings under different treatments.

Treatment/ Parameter	CK	NC	W11	W14
Height (cm)	8.15 ± 0.69c	9.11 ± 0.71b	10.89 ± 1.14a	11.28 ± 0.87a
SD (cm)	2.38 ± 0.36d	2.69 ± 0.26c	3.14 ± 0.26b	3.44 ± 0.16a
FW (g)	0.88 ± 0.17b	1.08 ± 0.31b	1.42 ± 0.28a	1.67 ± 0.25a
DW (g)	0.13 ± 0.04b	0.15 ± 0.04ab	0.18 ± 0.03ab	0.19 ± 0.04a
RL (cm)	14.29 ± 0.93c	15.03 ± 0.79bc	15.75 ± 0.67ab	16.84 ± 1.83a
RFW (g)	0.18 ± 0.09c	0.24 ± 0.05c	0.35 ± 0.05b	0.47 ± 0.09a
RDW (g)	0.06 ± 0.02b	0.08 ± 0.01ab	0.08 ± 0.02ab	0.10 ± 0.03a

NC, soil from a soybean rotation system; CK, soil under continuous sugar beet cropping for 2 years; W11, 2-year continuous sugar beet soil inoculated with strain W11; W14, 2-year continuous sugar beet soil inoculated with strain W14. Height, plant height; SD, stem diameter; FW, aboveground fresh weight; DW, aboveground dry weight; RL, root length; RFW, root fresh weight; RDW, root dry weight. Values are presented as mean ± standard deviation (SD) (*n* = 6). Different lowercase letters within the same row indicate significant differences among treatments (Tukey’s HSD test, *p* < 0.05).

### 3.2 Disease index

Disease index differed significantly among treatments (Figure 1B). The highest index was recorded under CK (54.59), followed by NC (31.76). In contrast, W11 and W14 substantially suppressed the disease, producing indices of 19.46 and 16.11, respectively. Both values were significantly lower than those under CK and NC, and W14 produced the lowest index. Therefore, complete genome sequencing of strains W11 and W14 was performed to explore potential genomic correlates of their different disease-control capacities.

### 3.3 Antagonistic effect

Figure 2 shows the dual-culture plate assays of strains W14 and W11 against *R. solani*. The inhibition rates of strains W14 and W11 were 77.72 and 64.05%, respectively. As shown in Figure 2A, strain W14 significantly inhibited *R. solani* mycelial growth. The pathogen mycelia were sparse and showed restricted growth on the plates. In contrast, the mycelia on the strain W11-treated plate were denser, indicating markedly weaker inhibition. Overall, the assays demonstrated that strain W14 exhibited significantly greater antagonistic activity against *R. solani* than strain W11.

**Figure 2:**
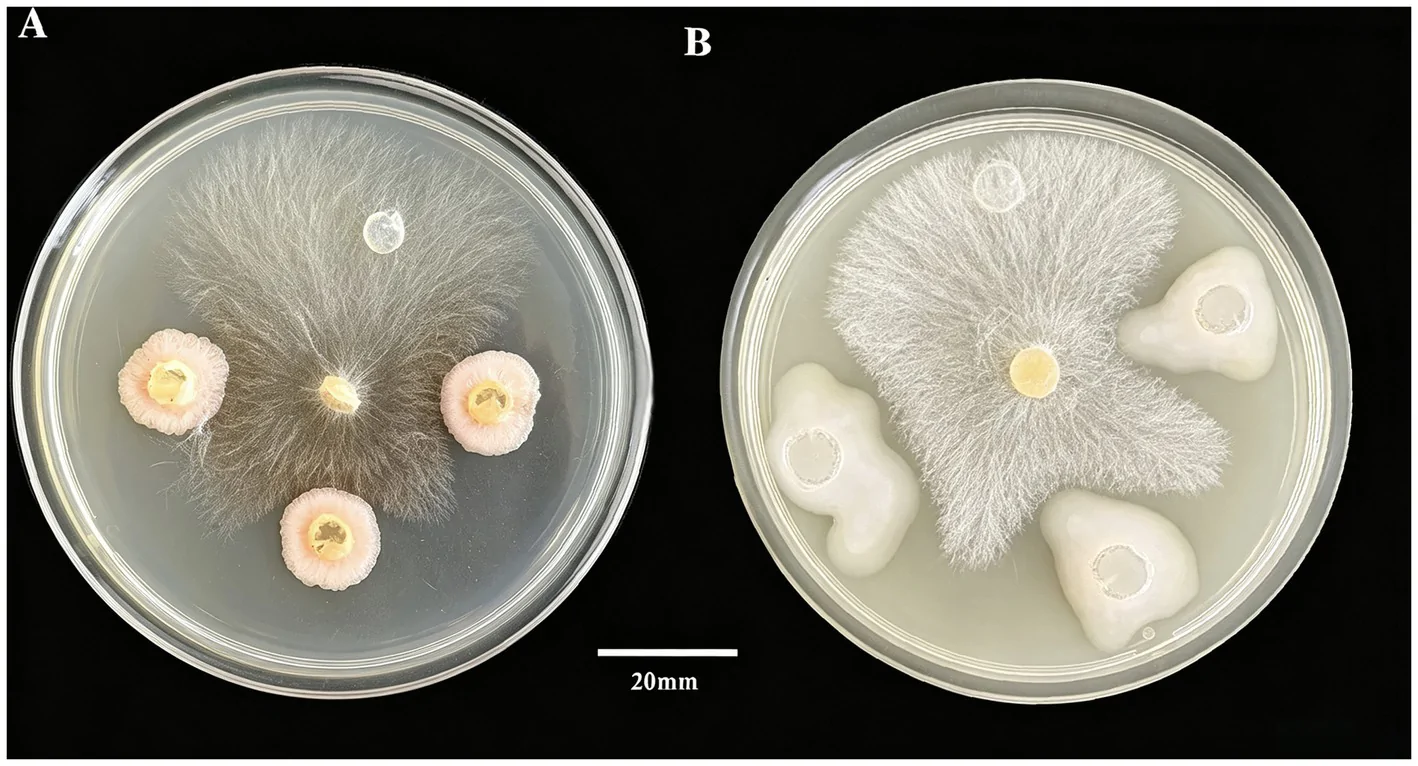
*In vitro* confrontation assay between myxobacterial strains and *Rhizoctonia solani*. **(A)** Plate confrontation assay between strain W14 and *Rhizoctonia solani*; **(B)** plate confrontation assay between strain W11 and *Rhizoctonia solani*.

### 3.4 Comparison genomic analysis

#### 3.4.1 Genomic maps

Comparative circular plots were generated for strains W14 and W11, each with a genome size of 12.3 Mb (Figure 3). The strains differed markedly in their number of chitinase genes. Although strain W14 (*C. ferrugineus*) harbored six GH19 chitinase genes and four GH5 glucanase genes (Figure 3A), strain W11 (*C. fuscus*) contained only three GH19 chitinase genes and two GH5 glucanase genes (Figure 3B). This expansion of cell wall-degrading enzyme genes in strain W14 may be associated with its greater biocontrol efficacy against Rhizoctonia blight, although functional validation is required.

**Figure 3:**
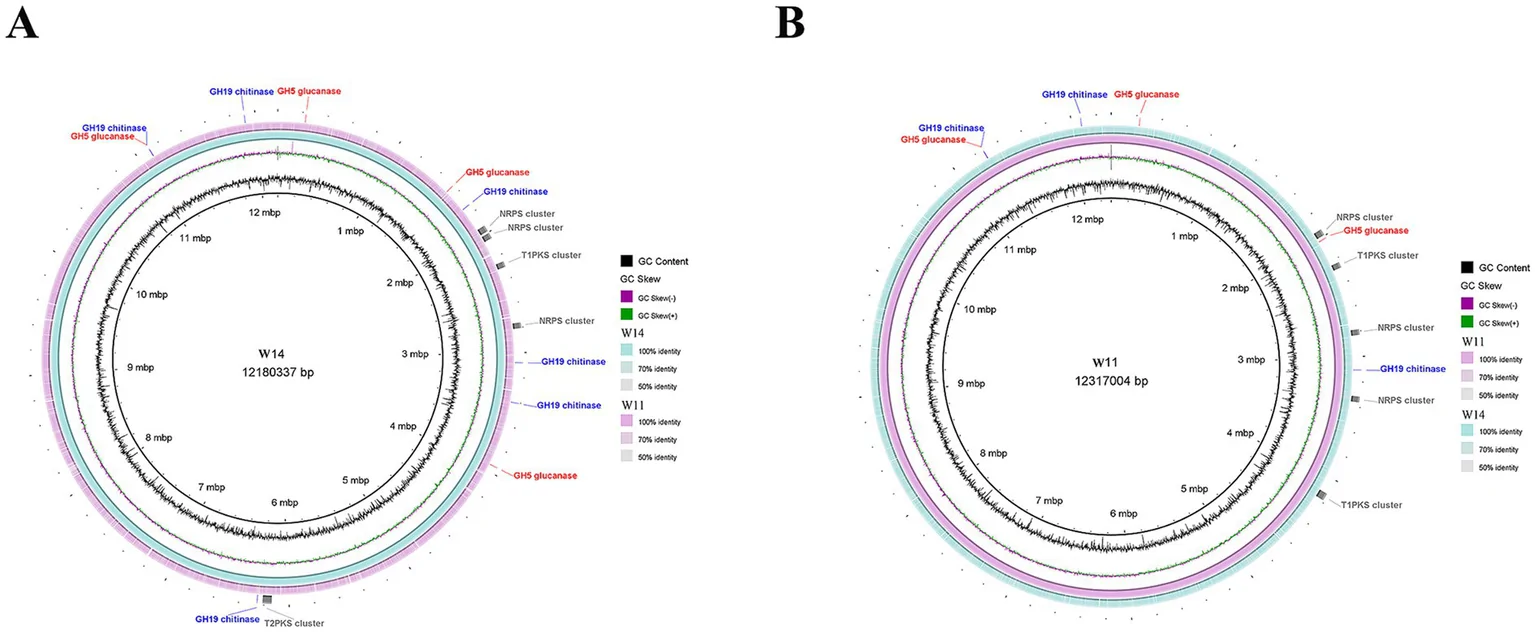
Circular genome maps of the two myxobacterial strains. **(A)** Circular genome map of strain W14; **(B)** circular genome map of strain W11. From the outermost to the innermost rings, the maps show BLAST-based synteny alignment, secondary metabolite biosynthetic gene clusters (gray), GH19 chitinase genes (blue), GH5 glucanase genes (red), GC skew, and GC content.

The circular alignment based on BLAST indicated high overall collinearity between the genomes, although strain W14 generally had a higher sequence similarity to the reference genome than strain W11. Strain W14 also contained a larger and more diverse set of secondary metabolite biosynthetic gene clusters, including T2PKS clusters, which suggests a greater potential to synthesize antimicrobial compounds. The GC content and GC skew profiles in the innermost rings confirmed the complete genome assemblies.

#### 3.4.2 NR annotation, GO classification, and KEGG pathway analysis

The NR annotation assigned the vast majority of genes from both strains to *Cystobacter*. Of the annotated genes in strain W11, 94.24% matched *Cystobacter fuscus*, supporting its taxonomic classification (Figure 4A, right panel). In strain W14, 74.52% of annotated genes also matched *C. fuscus*, whereas only 11.53% matched *Cystobacter ferrugineus* (Figure 4A, left panel). These results suggest that both strains have a higher sequence similarity to the *C. fuscus* reference genome, reflecting their close phylogenetic relationship. The remaining annotated sequences were similar to those of other myxobacterial genera, including *Corallococcus*, *Melittangium*, and *Archangium*. This finding indicates extensive gene conservation across Myxococcales.

**Figure 4:**
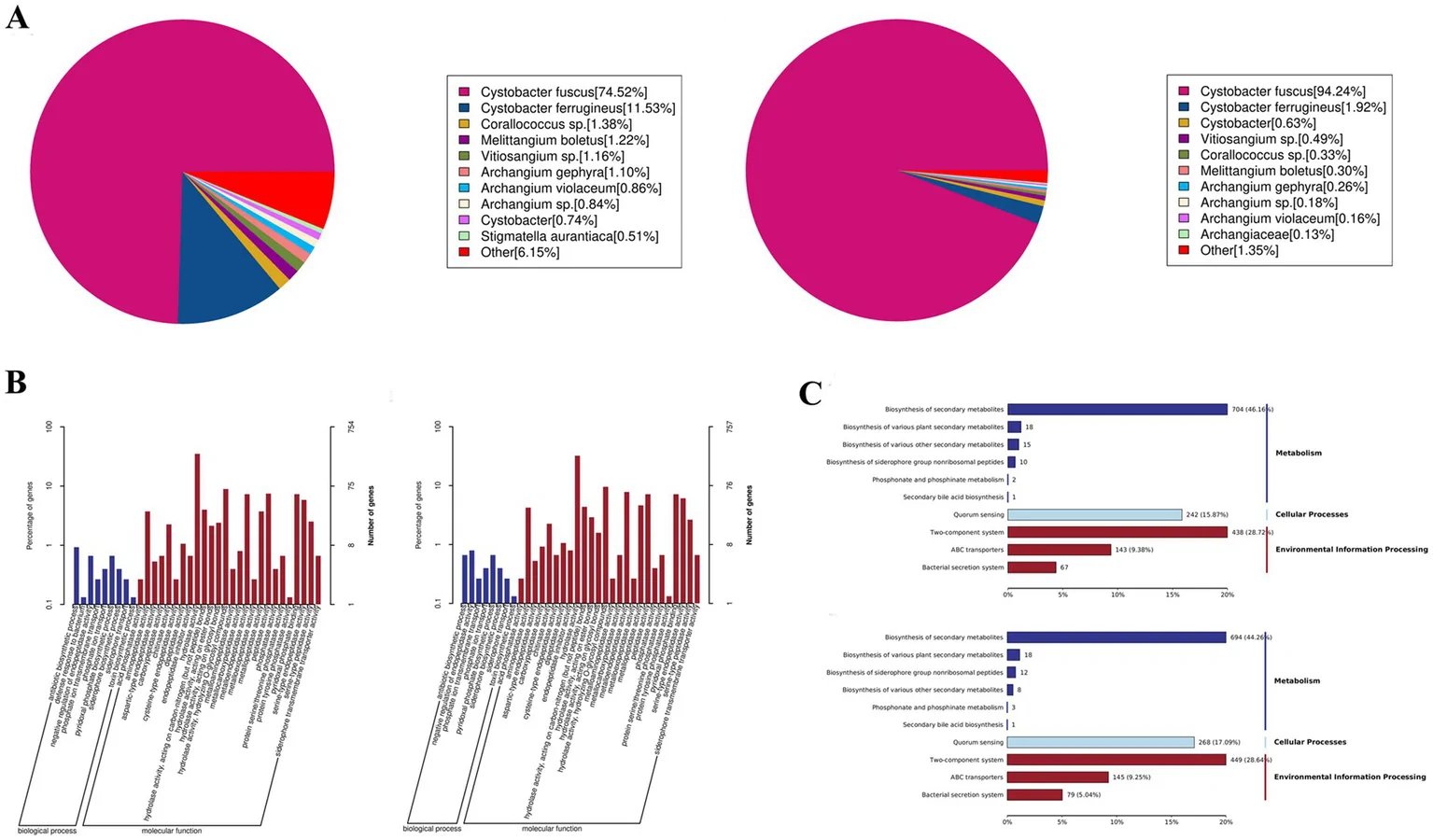
Comparison of NR annotation, GO second-level functional classification, and KEGG pathway annotation between strains W14 and W11. **(A)** Taxonomic distribution of NR annotations; **(B)** Comparison of GO second-level functional classification between strains W14 and W11; **(C)** Comparison of KEGG pathway annotations between strains W14 and W11. For **(A–C)**, the left and right panels represent strains W14 and W11, respectively.

To evaluate the genetic basis of biocontrol potential, we grouped annotated genes into functional categories related to disease resistance. Across the principal categories of cell wall degradation, antibiotic synthesis, secretion systems, toxin production, siderophore biosynthesis, and signal transduction, the two strains had comparable gene numbers (Table 3). Conversely, strain W14 contained slightly more genes than strain W11 in three critical categories: cell wall degradation, antibiotic synthesis, and signal transduction. Together, these differences suggest that strain W14 may have greater genomic potential for direct pathogen antagonism, antimicrobial compound production, and regulation of defense responses.

**Table 3:** Comparison of disease resistance-related functional gene categories between strains W14 and W11.

Category	Strain W14 count	Strain W11 count
Cell wall degradation	230	223
Antibiotic synthesis	31	28
Secretion system	88	93
Toxin production	12	12
Siderophore biosynthesis	32	33
Signal transduction	166	154
Other	9,130	9,328

Gene numbers were derived from NR database annotations. Functional categories were manually assigned based on annotation results.

The GO classification at the second level revealed highly similar functional distributions in the two strains, with most genes assigned to Biological Process and Molecular Function categories (Figure 4B). Strain W14 contained significantly more genes associated with hydrolase activity than strain W11, particularly in the subcategory of hydrolase activity acting on glycosyl bonds. In addition, strain W14 exhibited certain advantages over strain W11 in terms of genes associated with peptidase and metallocarboxypeptidase activities, suggesting a stronger proteolytic capacity. Moreover, the larger number of antibiotic biosynthesis genes in strain W14 was consistent with its richer complement of secondary metabolite biosynthetic gene clusters and indicated a greater potential for antimicrobial compound production. Defense response-related genes were annotated only in strain W14, which may contribute to its biocontrol potential. Overall, although both strains had highly similar broad functional profiles, GO analysis demonstrated that strain W14 had a more favorable gene composition than strain W11 in key disease resistance-related categories, providing candidate genomic correlates of its greater biocontrol efficacy against *R. solani*.

KEGG pathway annotation identified genomic differences between the two myxobacterial strains at the metabolic network level (Figure 4C). In the biosynthesis of other secondary metabolite pathways, strain W14 contained 15 annotated genes, approximately twice the eight genes in strain W11, which could corroborate the higher abundance of secondary metabolite biosynthetic gene clusters in the W14 genome. In the biosynthesis of siderophore group nonribosomal peptide pathway, strain W11 contained 12 genes, which was slightly more than the 10 in strain W14. This difference indicates that strain W11 may have a moderate advantage in siderophore production. This served as a feature potentially associated with iron competition-mediated antagonism and growth promotion in the rhizosphere. Furthermore, the strains carried comparable numbers of genes in the two-component system, ABC transporters, and quorum sensing pathways, which could be associated with signal transduction and substance transport.

In summary, strain W14 had a more extensive genetic complement in pathways related to secondary metabolism, whereas strain W11 had a marginal advantage in the siderophore biosynthesis pathway. Both strains shared a fundamentally similar genetic framework for basic signal transduction and substance transport.

#### 3.4.3 CAZy and PHI annotation

Specialized database annotation revealed molecular functional differences in antagonism between the strains. According to the CAZy analysis (Figure 5A), strain W14 contained twice as many GH19 family chitinase genes as strain W11, although both strains carried nine GH18 family chitinase genes. Additionally, strain W14 possessed three more GH5 family glucanase genes than strain W11. Chitinases (GH19/GH18) and glucanases (GH5) are key enzyme systems that degrade chitin and β-glucan, the core components of fungal cell walls. In contrast, strain W11 contained six more CBM50 family genes, whose products typically bind chitin or peptidoglycan. Gene counts were largely comparable between the strains for the remaining families, including GH16, GH30, GH55, AA10, CBM5, CBM12, and GH23.

**Figure 5:**
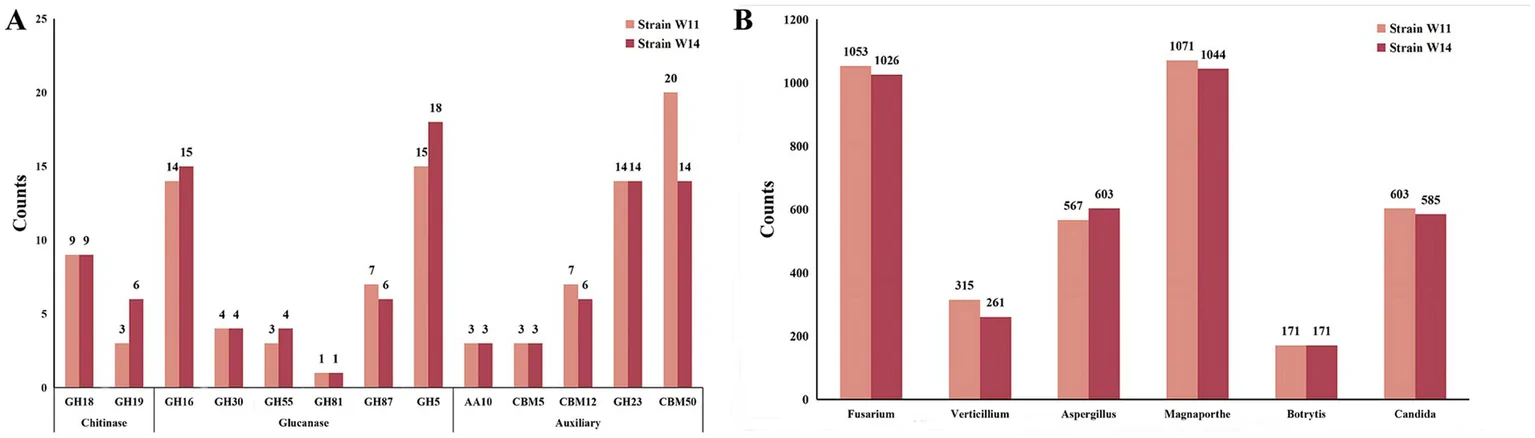
Comparison of CAZy and PHI annotations between strains W14 and W11. **(A)** Comparison of CAZy annotations; **(B)** Comparison of PHI annotations.

Analysis of the PHI database identified differences between the strains in the distribution of homologous genes associated with interactions between plants and pathogenic fungi (Figure 5B). Strain W14 was significantly enriched in the homologs of pathogenicity-related genes associated with *Aspergillus*, while strain W11 carried more homologs related to *Fusarium*, *Verticillium*, *Magnaporthe*, and *Candida*. Both strains contained identical numbers of homologs related to *Botrytis*. Although strain W11 contained more PHI-derived homologs targeting several fungal pathogens, strain W14 achieved superior disease control in the pot experiment. This apparent discrepancy suggests that PHI homolog counts alone are not predictive of biocontrol efficacy and that gene expression, protein activity, and substrate specificity may also be important.

In summary, CAZy analysis showed that strain W14 had an advantage in GH19 and GH5 family genes encoding cell wall-degrading enzymes, whereas PHI analysis identified more homologs associated with various pathogenic fungi in strain W11. Nevertheless, the latter advantage did not confer stronger disease control in the pot experiments.

#### 3.4.4 antiSMASH secondary metabolite analysis

To explore potential genomic correlates of the differences in biocontrol potential between the myxobacterial strains, antiSMASH was used to predict the secondary metabolite biosynthetic gene clusters (BGCs) in their genomes, focusing particularly on clusters associated with nonribosomal peptide synthetases (NRPS) and polyketide synthases (PKS). Figure 6 illustrates that strain W14 contained four NRPS/PKS gene clusters: an NRPS-like cluster (R31, 80% similarity to minimycin/indigoidine), a hybrid NRPS-T1PKS cluster (R32, 63% similarity to the antifungal compound myxalamid), a T1PKS cluster (R34) sharing only 30% similarity with the stigmatellin biosynthetic gene cluster, and an NRPS cluster (R40, 91% similarity to myxochelin). No product can be assigned to R34 based on this level of similarity. Conversely, strain W11 contained only two clusters, one T1PKS and one NRPS.

**Figure 6:**
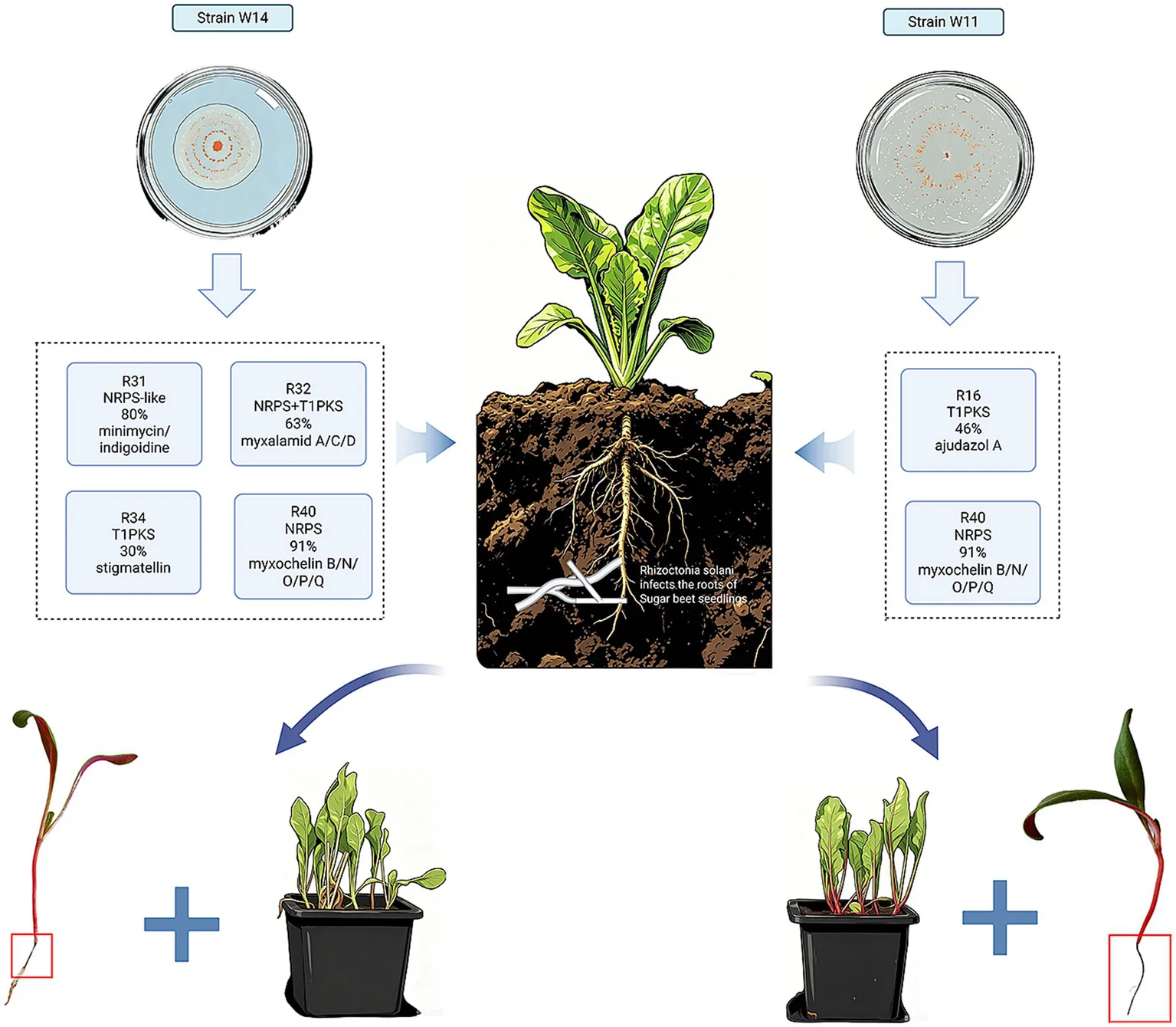
Proposed model illustrating the relationship between secondary metabolite biosynthetic gene clusters and biocontrol efficacy against *Rhizoctonia solani*. The left and right panels illustrate the plant growth-promoting and disease control effects of strains W14 and W11, respectively. The red boxes indicate disease symptoms in sugar beet seedling roots, which were less severe in the W14 treatment than in the W11 treatment.

It should be noted that antiSMASH predictions are based on sequence similarity and conserved domain architecture. A predicted BGC with low similarity to known clusters may represent a novel BGC, a silent or non-functional cluster, or a false-positive prediction. Therefore, the presence of R34 does not provide evidence for the actual production of stigmatellin or related compounds. Functional validation, such as gene knockout, heterologous expression, transcriptomic analysis, and metabolomics, is required to determine whether R34 is functional and whether it contributes to antagonism.

The number and diversity of NRPS/PKS gene clusters were markedly greater in strain W14 than in strain W11. Notably, the hybrid NRPS-T1PKS cluster R32 in strain W14 suggests the potential to synthesize antifungal secondary metabolites with more complex structures and higher functional diversity. Myxalamid is a known myxobacterium-derived antifungal compound, whereas minimycin and myxochelin are associated with antibacterial and siderophore-related functions, respectively. The enrichment of these clusters may provide candidate genetic evidence for the antagonistic potential of strain W14.

Although strain W14 contained more NRPS/PKS gene clusters than strain W11, some clusters, including R34, exhibited relatively low similarity (30%) to known compounds. Therefore, R34 represents a candidate BGC with an unknown product, and whether it is functional or produces a novel antifungal compound requires experimental validation. antiSMASH predictions should be interpreted as genomic predictions rather than evidence of actual metabolite production.

## 4 Discussion

Biological control has increasingly become an important alternative or complement to chemical methods. Using pot experiments, the present study evaluated the growth-promoting and biocontrol effects of two myxobacterial strains previously selected in our laboratory, *Cystobacter ferrugineus* W14 and *Cystobacter fuscus* W11, on sugar beet seedlings. Comparative genomic analysis was performed to explore the potential genomic correlates underlying their different biocontrol capacities.

### 4.1 Growth promotion of sugar beet seedlings by the two myxobacterial strains

Compared with CK, inoculation with strains W14 or W11 significantly increased sugar beet seedling growth parameters, including plant height, stem diameter, aboveground fresh and dry weights, and root fresh and dry weights. Plant height and stem diameter exceeded those under the rotation treatment (NC), indicating strong growth-promoting potential. Multiple mechanisms may account for this effect. First, both strains produced siderophores, which served as a capability facilitated myxobacterial motility and enhanced predatory activity (Contreras-Moreno et al., 2024), while also improving plant nutrition and nutrient uptake efficiency by modulating interactions within the plant–soil microecological system (Wang et al., 2024; Wang T. Q. et al., 2025). Second, the strains had similar genetic complements in signal transduction and substance transport pathways, including two-component systems and ABC transporters. This could provide a fundamental basis for stable rhizosphere colonization and the performance of growth-promoting functions.

Regarding disease control, strains W14 and W11 significantly reduced the disease index of sugar beet Rhizoctonia blight by 70.49 and 64.35%, respectively, compared with the CK, and both were more effective than the rotation treatment. These results are consistent with those of previous studies. Han et al. identified a *Cystobacter fuscus* strain with broad-spectrum antifungal activity against several phytopathogenic fungi, including *Verticillium dahliae* and *Fusarium oxysporum*, which controlled cotton Verticillium wilt by 70.9% under pot conditions (Han et al., 2025). Wang J. H. et al. (2025) reported that *Myxobacteria* MP20 stabilized antifungal factors through outer membrane vesicles (OMVs), thereby effectively inhibiting *V. dahlia*. Together, these findings indicate that myxobacteria can markedly promote sugar beet seedling growth and reduce seedling disease incidence.

### 4.2 Genomic basis of differences in biocontrol between the myxobacterial strains

Genetic differences may contribute to variation among biocontrol strains in metabolite biosynthesis, and these metabolites may influence their inhibitory capacity against phytopathogens (Zou et al., 2025). In the present study, strain W14 (*C. ferrugineus*) was significantly more effective in controlling sugar beet disease caused by *R. solani* than strain W11 (*C. fuscus*). Comparative genomic analysis explored potential genomic correlates for the difference in biocontrol capacity.

The expansion of GH19 genes in strain W14 may contribute to enhanced cell wall-degrading potential, and may represent one of the candidate molecular correlates of its greater antagonistic activity against *R. solani*. CAZy analysis identified six GH19 chitinase genes in strain W14, which was twice the number detected in strain W11. In contrast, each strain possessed nine GH18 chitinase genes. As a major structural component of fungal cell walls, chitin consists of an N-acetyl-D-glucosamine polymer whose β-1,4-glycosidic bonds are hydrolyzed by chitinases, disrupting cell wall integrity and causing pathogen death (Guo et al., 2020; Xu et al., 2022; Xiong et al., 2023). Since GH18 and GH19 both belong to the chitinase family, the difference in GH19 copy number does not necessarily translate into a significant difference in total chitinase activity. GH18 and GH19 may also differ in substrate specificity, catalytic mechanism, and expression regulation, and increased copy number does not necessarily lead to higher transcript or protein levels. The actual enzyme activity levels and substrate preferences remain to be validated by biochemical experiments. A mechanistically similar process has been reported in which myxobacteria degrade fungal cell walls using the outer membrane β-1,6-glucanase GluM (Zhang et al., 2026).

The enrichment of secondary metabolite biosynthetic gene clusters may provide strain W14 with an additional potential mechanism. The antiSMASH analysis identified four NRPS/PKS gene clusters in strain W14, including one hybrid NRPS-T1PKS cluster and one NRPS-like cluster, but only two such clusters In strain W11. Moreover, KEGG analysis confirmed that significantly more genes (15) in strain W14 than genes (8) in strain W11 were enriched in the biosynthesis of other secondary metabolite pathways. Myxobacteria can employ secondary metabolites as important chemical weapons against other microorganisms (Zhao et al., 2021). Similar to other myxobacteria, strain W14 has the potential to produce diverse active antifungal substances through its enriched complement of secondary metabolite gene clusters (Wang J. H. et al., 2025), mounting a “chemical attack” on pathogens.

### 4.3 Resolving the apparent phenotype–genotype discrepancy

The present study also presented some inconsistent results. First, the NR annotation indicated that 74.52% of genes in strain W14 matched *C. fuscus* and only 11.53% matched *C. ferrugineus*, demonstrating the high genomic similarity between the strains. Second, the PHI analysis identified more homologs associated with several fungal pathogens, including *Fusarium*, *Verticillium*, and *Magnaporthe*, in strain W11 than in strain W14. Nevertheless, strain W14 performed better than strain W11 in pot experiments.

Several factors may explain this paradox. The number of homologous genes in the PHI database is not directly equivalent to biocontrol capacity. The PHI annotations indicate only sequence similarity, whereas functional efficacy relies on factors such as gene expression regulation, protein activity, and substrate specificity. Although strain W11 carried more sequences that “mimic pathogenicity-related genes” of fungal pathogens, these genes failed to directly contribute to fungicidal activity. In contrast, strain W14 may concentrated its genomic resources in two core weapon systems, including chitinases through GH19 expansion and secondary metabolism through NRPS/PKS cluster enrichment. It is important to note that although GO, KEGG, CAZy, PHI, and antiSMASH represent different annotation strategies, they are all based on the same genomic sequence. Therefore, their findings should be interpreted as complementary genomic predictions rather than independent multi-omics validations. These predictions highlight candidate genomic correlates that require further functional validation. This may allow more efficient inhibition of pathogens through the combined action of “direct enzymatic degradation” and “secondary metabolite-mediated antimicrobial activity”. Furthermore, the lack of standardized artificial inoculation limits the direct comparison of disease-control efficacy across studies, highlighting the need for future validation using controlled pathogen challenge assays.

## 5 Conclusions and perspectives

In summary, both myxobacterial strains promoted sugar beet seedling growth and reduced disease under naturally infested continuous-cropping soil. Comparative genomic analysis suggested that strain W14 may possess a potential dual biocontrol strategy of “cell wall degradation + antimicrobial compound synthesis” through the expansion of GH19 chitinase genes and enrichment of secondary metabolite biosynthetic gene clusters. These genomic annotations provide candidate correlates rather than definitive determinants of biocontrol efficacy, and may offer a candidate molecular basis for its stronger antagonism against *R. solani*. This study identified candidate genes and provides candidate strains and functional gene clues for the development of *C. ferrugineus*-based biocontrol agents, rather than establishing a scientific foundation for agent development.

However, this study has several limitations. (i) Experimental conditions: only one sugar beet variety (‘KWS1260’) was tested, no standardized artificial inoculation was performed, and root yield, sucrose content, and systematic before-and-after disease photographs were not evaluated. The initial pathogen load and spatial heterogeneity in naturally infested soil could not be fully controlled, which may affect reproducibility. (ii) Genomic analysis: only one strain of each species was sequenced, and the genomic predictions were not functionally validated; low-similarity BGCs such as R34 require experimental validation. (iii) Interpretive scope: because of these limitations, the genomic differences should be interpreted as candidate correlates rather than established determinants of biocontrol efficacy, and the current data only support tentative associations between genomic features and biocontrol phenotypes.

Future studies should combine standardized artificial inoculation, randomized block designs, and multi-location trials with multiple sugar beet varieties, evaluate root yield, sucrose content, soil colonization, survival, and optimal application conditions, compare efficacy with commercial biocontrol strains such as *Bacillus* and *Trichoderma*, and validate key genes and metabolites through gene knockout, heterologous expression, transcriptomic analysis, and metabolomic profiling.

## Data Availability

The datasets presented in this study can be found in online repositories. The names of the repository/repositories and accession number(s) can be found at: https://www.ncbi.nlm.nih.gov/, PRJNA1499328.
