# Supplementary material for: Two myxobacterial strains against Rhizoctonia solani: comparative genomics of antagonistic potential and phenotypic evaluation of growth-promoting and disease-control effects
Source: Front Microbiol. 2026 Oct 14;17:1975171. doi: 10.3389/fmicb.2026.1975171 (PMC13713471; doi:10.3389/fmicb.2026.1975171)
Supplement: Supplementary file 1 [file Table_1.DOCX]

**1. Materials**

1.1 **Soil Collection**

Soil was collected from the Hulun Campus of Heilongjiang University (latitude and longitude: 46°00′14″E, 126°38′49″N). In each sampling area, a five-point sampling method was adopted: five points were selected along the diagonal of the sampling plot. After scraping off the surface soil at each point, soil samples at a depth of 10 cm were collected. The five subsamples were mixed thoroughly to obtain a composite sample, and 100 g of fresh soil was placed into a sterile self-sealing bag. After returning to the laboratory, the samples were immediately air-dried after removing stones and preserved dry at room temperature.

**1.2 Media Required for Experiments**

(1) WCX solid medium: CaCl_2_·2H_2_O 1 g/L, 3-morpholinopropanesulfonic acid (MOPS) 2.093 g/L, agar powder 15 g/L, cycloheximide solution at 25 μg/mL, pH 7.2.

(2) VY/2 solid medium: Angel yeast 5 g/L, CaCl_2_·2H_2_O 1 g/L, 3-MOPS 2.093 g/L, agar powder 15 g/L, pH 7.2; after sterilization, VB_12_ was added to a final concentration of 50 μg/mL.

(3) LB solid medium: LB liquid medium powder 25 g/L, agar 25 g/L.

(4) LB liquid medium: LB liquid medium powder 25 g/L.

**2. Isolation and Purification of Strains**

**2.1 Soil Pretreatment**

The air-dried soil was passed through a 40-mesh sieve to remove small stones and residual plastic mulch. Then, 15 g of the sieved soil was placed into a sterile Petri dish, and 20 mL of cycloheximide solution (200 μg/mL) was added for soaking for 12-24 h. After soaking, the liquid and soil in the dish were transferred to a 50-mL centrifuge tube and allowed to stand for 30 min for solid–liquid separation. The supernatant was removed as much as possible, and the soil pellet was retained.

**2.2 *Escherichia coli* Induction Method**

E. coli cultured on LB solid medium was inoculated into LB liquid medium and incubated overnight at 37℃ with shaking at 180 r/min. Then, 1 mL of the bacterial culture was transferred to a 1.5-mL centrifuge tube and resuspended. Using a pipette, 200 μL of the resuspended E. coli suspension was taken and spread with a sterilized glass spreader to form a spot of 2 cm in diameter on the WCX medium, and then air‑dried for later use. A glass rod was dipped into the bacterial suspension and used to draw a "cross", a "grid" (tic-tac-toe pattern), and three parallel lines on the WCX medium. The pretreated soil samples were placed respectively at the center of the bacterial spot, on a blank area, and at one end of the parallel lines. The plates were then incubated at 30℃ in a constant‑temperature incubator, and observed for the appearance of fruiting bodies after 10 days.

**2.3 Purification of Myxobacteria**

Single fruiting bodies appearing at the soil margin of the isolation medium were picked with a sterile toothpick tip under a stereomicroscope and spot-inoculated onto the surface of VY/2 medium, followed by incubation at 30 °C for 7 d.

**3. Strain Identification**

**3.1 Morphological Identification**

According to the taxonomic criteria for myxobacteria described in the eighth edition of Bergey's Manual of Determinative Bacteriology, the morphology and color of fruiting bodies as well as colony morphology were observed and recorded. After Gram staining, cell morphology was examined. Based on these characteristics, a preliminary identification was conducted to assign the isolated strains to the genus level.

**3.2 PCR Amplification of Myxobacterial 16S rRNA Gene**

Fruiting bodies at the edge of colonies were picked with sterile toothpicks and transferred to sterile centrifuge tubes. A small amount of quartz sand was added for grinding, and genomic DNA of the strains was extracted using the Bacterial Genomic DNA Extraction Kit (BL1044A). Using the extracted DNA as template, PCR amplification was performed with the universal bacterial primers 27F (5´-AGAGTTTGATCCTGGCTCAG-3´) and 1492R (5´-TACGGCTACCTTGTTACGACTT-3´). The PCR reaction system (50 μL) consisted of: 1 μL of strain DNA, 1 μL of primer 27F, 1 μL of primer 1492R, 25 μL of Mix enzyme, and 22 μL of ddH₂O. The amplification conditions were as follows: initial denaturation at 94℃ for 5 min; 30 cycles of denaturation at 94℃for 30 s, annealing at 55℃ for 45 s, and extension at 72℃for 1.5 min; followed by a final extension at 72℃ for 10 min.

**3.3 Gel Electrophoresis Analysis of PCR Products and Sequencing**

The amplified PCR products were subjected to agarose gel electrophoresis and photographed using an imaging system. Strains showing an electrophoresis band at approximately 1500 bp were sent to Sangon Biotech (Shanghai) for sequencing. The obtained sequences were subjected to BLAST alignment on the NCBI website, and a phylogenetic tree was constructed.

**4. Results and Analysis**

Morphological observation of the myxobacteria was performed for preliminary identification. On VY/2 solid medium, the myxobacteria exhibited distinct morphological characteristics. Strain W11 grew radially on VY/2 solid medium, forming a distinct translucent membrane with a relatively thin texture; its fruiting bodies were orange and spread in concentric rings. Microscopic observation revealed that the fruiting bodies were spherical or ellipsoidal, orange in color, with mature fruiting bodies remaining orange (Figure S1A, S2A). Strain W14 produced brown fruiting bodies on VY/2 solid medium, which also spread in concentric rings; microscopically, the fruiting bodies were spherical or ellipsoidal, orange, with mature fruiting bodies appearing brownish-orange (Figure S1B, S2B). These characteristics were consistent with the morphological descriptions of myxobacteria (Myxobacterales) in Bergey's Manual of Determinative Bacteriology.

Using the genomic DNA extracted with the kit as template, PCR amplification was performed with the universal bacterial primers 27F and 1492R. The products were analyzed by 1% agarose gel electrophoresis. As shown in Figure S3, clear and bright single bands were observed at approximately 1500 bp, indicating that the amplified fragments corresponded to the target gene fragments of the myxobacterial strains.

Based on BLAST homology comparison of the 16S rRNA gene sequences (the sequences of strains W11 and W14 have been submitted to GenBank under accession numbers QB034646 and QB034649, respectively), strain W11 showed the highest sequence similarity to *Cystobacter fuscus*, ranging from 97.41% to 100%, while strain W14 showed the highest sequence similarity to *Cystobacter ferrugineus* Cb fe27, reaching 100%. A Maximum Likelihood phylogenetic tree was constructed using MEGA 12.0 (Figure S4). Strain W11 clustered with *Cystobacter fuscus*, and strain W14 clustered with *Cystobacter ferrugineus*.

**Figure Captions**

**Figure S1** Colony morphology of strains W11 and W14. (A) Colony of strain W11; (B) Colony of strain W14.

**Figure S2** Microscopic observation of fruiting bodies of strains W11 and W14. (A) Fruiting bodies of strain W11; (B) Fruiting bodies of strain W14. Both were observed at 10× magnification.

**Figure S3** Agarose gel electrophoresis results of PCR-amplified products. Lane 11: strain W11; Lane 14: strain W14.

**Figure S4** Phylogenetic tree of strains based on 16S rRNA gene sequences.
